# Lactate Receptor HCAR1 Affects Axonal Development and Contributes to Lactate’s Protection of Axons and Myelin in Experimental Neonatal Hypoglycemia

**DOI:** 10.1523/ENEURO.0563-24.2025

**Published:** 2025-05-30

**Authors:** Lauritz Kennedy, Cecilie Morland, Martine Narum, Linda H. Bergersen, Johanne E. Rinholm

**Affiliations:** ^1^Department of Microbiology, Oslo University Hospital and University of Oslo, Oslo 0373, Norway; ^2^ Division of Physiology, Institute of Basic Medical Sciences, University of Oslo, Oslo 0372, Norway; ^3^ Department of Pharmacy, The Faculty of Mathematics and Natural Sciences, University of Oslo, Oslo 0371, Norway; ^4^ The Brain and Muscle Energy Group, Institute of Oral Biology, Faculty of Dentistry, University of Oslo, Oslo 0372, Norway; ^5^King Abdullah University of Science and Technology, Kaust Smart-Health, Thuwal 23955, Saudi Arabia

**Keywords:** axonal development, development, HCAR1, hypoglycemia, lactate, myelin

## Abstract

Lactate plays an important role in brain energy metabolism. It contributes to normal brain development and to neuroprotection in diabetic hypoglycemia, but its role in neonatal hypoglycemia is unclear. Moreover, lactate can work as a signaling substance via the lactate receptor HCAR1 (Hydroxycarboxylic acid receptor 1). Recent studies indicate that HCAR1 is protective in mouse models of neonatal hypoxic ischemia and has a role in metabolic regulation in glial cells during hypoglycemia. Here we have studied potential impacts of HCAR1 on axonal and myelin development in the cerebral cortex and corpus callosum of young (P21) wild-type (WT) mice and HCAR1 KO mice and in cortical organotypic brain slice cultures. The HCAR1 KO mice showed lower axonal area relative to WT in both cortex and corpus callosum. However, the myelin area was unaffected by HCAR1 KO. Using particle and colocalization analysis, we show that HCAR1 KO predominantly reduces axonal size in unmyelinated axons. Using an organotypic brain slice model of neonatal hypoglycemia, we find that lactate protects both axonal and myelin development in hypoglycemia, partially via HCAR1. Lastly, live imaging with a pH-sensitive dye on acute cortical brain slices indicates that cellular lactate uptake is influenced by HCAR1. In conclusion, our findings support a role of HCAR1 in axonal development and in lactate’s protective effects in hypoglycemia.

## Significance Statement

Lactate is a critical metabolite for brain energy metabolism, with established roles in neuroprotection and development. Our study provides new insights into the role of the lactate receptor HCAR1 in axonal and myelin development in the neonatal brain. We demonstrate that HCAR1 influences axonal size, particularly in unmyelinated axons, and mediates lactate’s protective effects during neonatal hypoglycemia. Using in vivo and ex vivo approaches, including organotypic brain slice cultures and live imaging, we show that HCAR1 influences cellular lactate uptake and protects axonal and myelin integrity under hypoglycemic conditions. These findings highlight the dual role of lactate as an energy substrate and signaling molecule via HCAR1, with implications for understanding brain development and resilience to metabolic stress.

## Introduction

Metabolic pathways are closely linked to biological processes such as cell signaling, differentiation, and proliferation and are thereby vital regulators of neurodevelopment (Miyazawa and Aulehla, 2018; Steiner, 2019). The small metabolite lactate is now well established as an energy substrate for neurons (Pellerin, 1998; Schurr, 2006; Fünfschilling et al., 2012) and brain lactate concentration increases during early human cerebral development (Tomiyasu et al., 2016). Further, lactate metabolism is associated with cell proliferation (Warburg, 1956; Boland et al., 1993; Vander Heiden et al., 2009) and brain maturation (Bauernfeind et al., 2014) including neural progenitor proliferation and survival (Gershon et al., 2013; Zheng et al., 2016). Additionally, lactate shows promise as a protector in traumatic brain injury (Mosaed et al., 2022), neonatal hypoxic ischemia (Tassinari and de Fraga, 2021), and diabetic hypoglycemia. In the latter, lactate can prevent cerebral dysfunction by contributing to energy metabolism and improve both hormone and symptom responses to hypoglycemia (Maran et al., 1994; Veneman et al., 1994; De Feyter et al., 2013). The dual role of lactate in cerebral development and hypoglycemic protection could be highly relevant in neonatal hypoglycemia, a common disease that causes acute brain injury and is thought to have long-term impacts on brain development (Duvanel et al., 1999; De Angelis et al., 2021).

Lactate has within the recent decade also been proposed as a signaling molecule in the brain (Bergersen and Gjedde, 2012; Mosienko et al., 2015) and in 2014 the G-protein coupled lactate receptor Hydroxycarboxylic acid receptor 1 (HCAR1, previously known as GPR81) was found to be expressed in the brain (Lauritzen et al., 2014). Since then it has been implicated in neuronal excitability and firing (Bozzo et al., 2013; Briquet et al., 2022), developmental angiogenesis (Chaudhari et al., 2022), and exercise-induced angio- and neurogenesis (Morland et al., 2017; Lambertus et al., 2021), as well as reduction of hypoxic-ischemic cell death in adult and neonatal mice (Castillo et al., 2015; Chaudhari et al., 2022; Geiseler et al., 2024). In line with this, HCAR1 protects against neonatal hypoxic-ischemic brain injury in mice through activation of thousands of genes involved in cell cycle, immune response, and neurogenesis (Kennedy et al., 2022). HCAR1 can also regulate astrocyte glycogen accumulation and breakdown in normo- and hypoglycemic conditions (Roy et al., 2023).

Axonal growth is crucial for brain development. In this process, neurons polarize and extend their axons to assemble neuronal circuits. Axonal generation is two phased. First, thin axonal processes grow outward and move up to their target cell. Second, radial expansion ensures axon thickness for fast and powerful excitations (Goldberg, 2003). In the first phase, the elongating axon is guided by attracting and repulsive stimuli on growth cone receptors that trigger intracellular impulses to push the axon in the right direction (O’Donnell et al., 2009; Kolodkin and Tessier-Lavigne, 2011). Several g-protein coupled receptors are involved in the guidance of axons (Xiang et al., 2002; O’Donnell et al., 2009), and a recent study showed that HCAR1 activation stimulates axonal growth in retinal explants from mice (Laroche et al., 2021).

Neonatal hypoglycemia is a common condition, which can lead to neurological injury when severe or recurrent (De Angelis et al., 2021). It is recommended that plasma glucose levels in the newborn infant stay above 3.3 mM with a somewhat wider tolerance during the first 48 h after birth where many infants experience physiological transient hypoglycemia (Thornton et al., 2015). In vitro models have become essential tools for studying the molecular processes in neonatal hypoglycemia, particularly in the context of brain development and injury.

Here we investigate the role of HCAR1 in axonal development and myelination in normo- and hypoglycemic conditions. We show by quantitative immunofluorescence that P21 HCAR1 knock-out (KO) mice have decreased levels of the axonal marker neurofilament heavy chain (NF200) in the cortex and corpus callosum compared with WT mice. In contrast, we find no difference in myelin content between KO and WT mice, and no indications of altered myelin-to-axon ratio in electron micrographs. We have shown previously that lactate can rescue axon and myelin loss in hypoglycemia in organotypic mouse brain slices (Rinholm et al., 2011). Here we show that the effect of lactate is partially dependent on HCAR1 stimulation: since lactate could rescue hypoglycemic axons and myelin to a larger degree in WT than in KO mice. Finally, pH imaging of acute brain slices suggests that HCAR1 KO mice have a higher lactate uptake than WT slices.

## Materials and Methods

### Experimental design

Three experimental setups were used in this study. For detailed information about the individual setups, see specific subsections.

1. Perfusion fixation with paraformaldehyde (PFA) of postnatal day 21 (P21) HCAR1 KO and WT mice combined with immunohistochemistry, confocal microscopy, and electron microscopy.

2. Organotypic brain slice cultures of P8 HCAR1 KO and WT mice combined with immunohistochemistry and confocal microscopy.

3. Confocal live imaging of acute brain slices treated with a pH-sensitive dye from P19 HCAR1 KO and WT mice.

### Animals

Male and female HCAR1^+/+^ (WT) and HCAR1^−/−^ (HCAR1 KO) mice were used in the experiments (Ahmed et al., 2010). All experimental procedures were approved by the local and national Animal Research Authority. Procedures were conducted by FELASA C-certified operators and complied with national laws and institutional regulations governing the use of animals in research.

### Perfusion fixation and cryosectioning

P21 mice were anesthetized with a lethal peritoneal injection of pentobarbital before perfusion through the left ventricle with 4% PFA depolymerized in 0.1 M sodium phosphate buffer, pH 7.4 (NaPi), by a peristaltic pump (100 ml/10 min). Brains were stored 24 h for postfixation in 0.4% PFA in NaPi at 4°C, followed by 4 h of cryoprotection in 30% sucrose and then 24 h in 40% sucrose in NaPi at 4°C before 30-µm-thick coronal sections were cut from bregma 1.0 to −2.0 mm (Allen Mouse Brain Reference Atlas) on a freezing microtome. *n* = 4–5 mice in each group.

### Solutions for brain slice experiments

Organotypic brain slice experiments: slicing medium (25 mM HEPES buffer, pH 7.35, in Earle’s Balanced Salt Solution (EBSS); high glucose culture medium (50% MEM-GlutaMAX with HEPES, 25% EBSS, 25% horse serum, 36 mM glucose, 0.5% Penstrep, all from Life Technologies, 0.125% nystatin (Sigma Aldrich), 5 mM Tris buffer); low-glucose culture medium (50% DMEM-GlutaMAX without sugar added 2 mM NaHCO3, 2 mM Alanyl-glutamine, and 12 mM NaCl, 25% horse serum, 25% EBSS, 0.5% Penstrep, 0.125% nystatin, 5 mM Tris buffer). For live imaging of acute brain slices: slicing medium (124 mM NaCl, 26 mM NaHCO3, 1.25 mM NaH2PO3, 3 mM KCl, 1 mM MgCl2, in H2O); aCSF (124 mM NaCl, 26 mM NaHCO3, 1.25 mM NaH2PO3, 3 mM KCl, 1 mM MgCl2, 2 mM CaCl2, in H2O).

### Organotypic brain slice cultures

P8 mice were decapitated, and 230-μm-thick coronal slices were cut on a vibratome between bregma 1.4 mm and −0.6 mm and prepared for organotypic culturing as described previously (De Simoni and MY Yu, 2006; Kennedy and Rinholm, 2017). Slices were fixed in 4% PFA after 13 d in vitro. During the first 3 d, all treatment groups were cultured in high glucose culture medium and then changed to a treatment-specific culture medium. NaCl in EBSS was added to the media to compensate for changes in osmolality. The medium was changed every 2–3 d. Slices were fixed in 4% PFA in NaPi for 1 h on a shaker at room temperature (RT). In each treatment group, *n* = 10–16 slices were pooled from two independent experiments per genotype. Glucose measurements were done in medium after 24, 48, and 72 h without medium change to see how the glucose concentration would change over time. A small sample (200 µl) of medium was removed from the well and kept at −20°C until measuring. Unused medium from high glucose and low-glucose media were used as controls (0 h). Measurements were done using two different approaches: a Glucose-Glo Assay (Promega) and i-STAT1 glucose meter (Abbott). Both methods gave similar values.

### Immunohistochemistry

Samples were washed in PBS three times for 15 min, incubated in 10 mM citrate buffer, pH 8–8.7, at 85°C for 30 min for antigen retrieval, and then blocked and permeabilized with 10% fetal calf serum and 0.5% Triton for 4 h. Primary antibody incubation was carried out overnight on a shaker at room temperature. The following day, slices were washed 3 × 20 min and incubated with secondary antibodies for 3.5–5 h on a shaker at RT. After secondary antibody incubation, slices were washed 3 × 20 min. DAPI staining was done for 15 min, followed by wash 3 × 10 min, before mounting on Superfrost object glass using ProLong antifade. Cover glass thickness was 0.13–0.17 mm. The primary antibodies used were anti-myelin basic protein (Millipore #MAB 386, RRID: AB_94975, 1:300), anti-NF200 (Abcam #AB4680, RRID: AB_304560, 1:10,000), and anti-NeuN (Millipore #MAB377, RRID: AB_2298772, 1:500). The secondary antibodies used were Alexa Fluor 555 Goat Anti-Chicken (Life Tech. #A-21437, RRID: AB_1500593), Alexa Fluor 488 Goat Anti-Rat (Life Tech., #A-11006, RRID: AB_2534074), Alexa Fluor 488 Goat Anti-Mouse (Life Tech., #A-1104, RRID: AB_141371). All secondary antibodies were diluted 1:400. DAPI was used for nuclear staining (Sigma #D-9542, 1:5,000).

### Electron microscopy

Tissue samples from perfusion fixed mice were cryoprotected in glycerol and plunged into liquid propane cooled to −170°C by liquid nitrogen. The samples were immersed in a solution of anhydrous methanol and 0.5% uranyl acetate overnight at −90°C. The temperature was then raised stepwise in 4°C increments per hour from −90 to −45°C, where it was kept for the subsequent steps. The tissue samples were washed several times with anhydrous methanol to remove residual water and uranyl acetate and then infiltrated in the embedding resin Lowicryl HM20 stepwise from Lowicryl/methanol 1:2, 1:1, and 2:1 (1 h each) to pure Lowicryl (overnight). Polymerization was catalyzed by 360 nm ultraviolet light for 2 d at −45°C followed by 1 d at room temperature. Ultrathin sections (90 nm) were cut by a diamond knife on a Reichert Jung ultramicrotome and mounted on nickel grids with an adhesive pen (David Sangyo).

The ultrathin sections were contrasted in uranyl acetate (5%) and lead citrate (30%), before they were observed in a Philips CM100 electron microscope. Axonal diameter and myelin thickness were measured by a blinded observer in ImageJ. G-ratio measurements were calculated with the formula g-ratio = ø Axon / (ø Axon + Myelin).

### pH imaging

Monocarboxylate transporters (MCTs) cotransport lactate and H^+^; hence their activity can be monitored from the pH change they produce (Meredith et al., 2002). We used confocal imaging and the pH-sensitive dye SNARF1 to study pH changes evoked by application of lactate in acute slices from WT and HCAR1 KO mice. Coronal slices from the mouse cerebral cortex (P19 mice) were cut at a thickness of 225 μm on a vibratome in the slicing medium described above. Slices were incubated in 10 µM 5-(and-6)-carboxy SNARF-1, acetoxymethyl ester, acetate (SNARF-1-AM, Thermo Fisher), and 0.01% Pluronic F-127 (Sigma) dissolved in slicing medium for 45 min in a chamber containing 95% O_2_ and 5% CO_2_ and then washed in slicing medium for 45 min at RT. Live imaging was done with the Zeiss LSM700 system, using excitation wavelength at 488 nm and emission captured with lambda mode, splitting channels at 600 nm. While imaged, slices were superfused with aCSF (and other solutions as described below) at 37°C and bubbled with 95% O_2_ and 5% CO_2_. The treatment setup was as follows: artificial cerebrospinal fluid (aCSF) 5 min, 10 mM lactate 5 min, aCSF 10 min, 10 mM NH_4_Cl 5 min, aCSF 15 min. The intracellular pH change was measured as the maximum change in the ratio of [emitted light > 600 nm] / [emitted light < 600 nm] from a predicted baseline based on the trajectory of the graph before and after the treatments. Lactate uptake was defined as the maximum pH change of lactate treatment/NH_4_Cl-treatment (control acid). *n* = 7–8 from two independent experiments.

### Imaging and image analysis of immunolabeled samples

Confocal images of fixed tissue were obtained on a Zeiss Meta 510 confocal system. A 20× objective lens (numeric aperture 0.8) was used for all experiments. In the perfusion fixed mice, three sections from each mouse were analyzed. The first section was from bregma 0.75 and then the following sections were picked at 600 µm intervals caudally. From cortical areas that included motor and sensory cortex, three *z*-stacks of six optical sections per section were taken. For corpus callosum, one *z*-stack of three optical sections per section were analyzed. In slices from the organotypic brain slice cultures, three areas per slice were imaged, each as a *z*-stack of three optical sections. In the cortex we quantified axons and myelin over a volume of 0.036 mm^3^, corresponding to an average of ∼2.4% of the total adult neocortex (Schüz and Palm, 1989). Image analyses were performed with FIJI (ImageJ2) software. Before quantification of the labeling, we performed image segmentation using automatic thresholding with the most fitting of the Li (Li and Lee, 1993) and Otsu algorithms (Otsu, 1979; Liu and Yu, 2009). To remove background and noise, we used the “remove outliers” (*r* = 1–3). The particle analysis tool was used before analysis to threshold objects by size, removing axons and myelin sheaths perpendicular to the imaging plane and structures not identifiable as “strands” of axons and myelin. See example images of segmentation in Extended Data Figure 1-1*F*. Area was measured by the number of positive, i.e., immunoreactive, pixels over background, corresponding to the area of the labeled structure. The particle analysis tool was used to count whole objects, e.g., axons. Counting of axons and myelin sheaths in the corpus callosum and organotypic brain slice cultures was not possible due to the density and significant overlapping of labeled structures. Colocalization analyses were performed on segmented images using the “and” and “subtract” functions in the image calculator tool before measuring area. All image analyses from immunohistochemistry were performed automatically using java-scripts designed for each experiment, i.e., blinding the observer. For organotypic slice culture experiments, data were normalized to the control group for each experiment, due to significant variation between experiments independent of genotype (data not shown).

### Statistical analysis

The statistical analyses were performed in Microsoft Excel and GraphPad Prism 9.5. *p* values were calculated with Student’s *t* test. In the organotypic brain slice culture experiments, two-way ANOVA with Holm–Šídák test for multiple comparisons was used. The two-way ANOVA interaction analysis was statistically significant in both the NF200 (*p* = 0.006) and MBP (*p* < 0.001) analyses and indicates that the treatment effects are influenced by genotype. Results are presented with *p* values from the Holm–Šídák test for multiple comparisons. All graphs are shown with individual datapoints with bars representing the mean. Error bars represent the standard deviation of the mean.

## Results

### Axonal area is reduced in the cortex and corpus callosum of young HCAR1 KO mice

**Figure 1.**
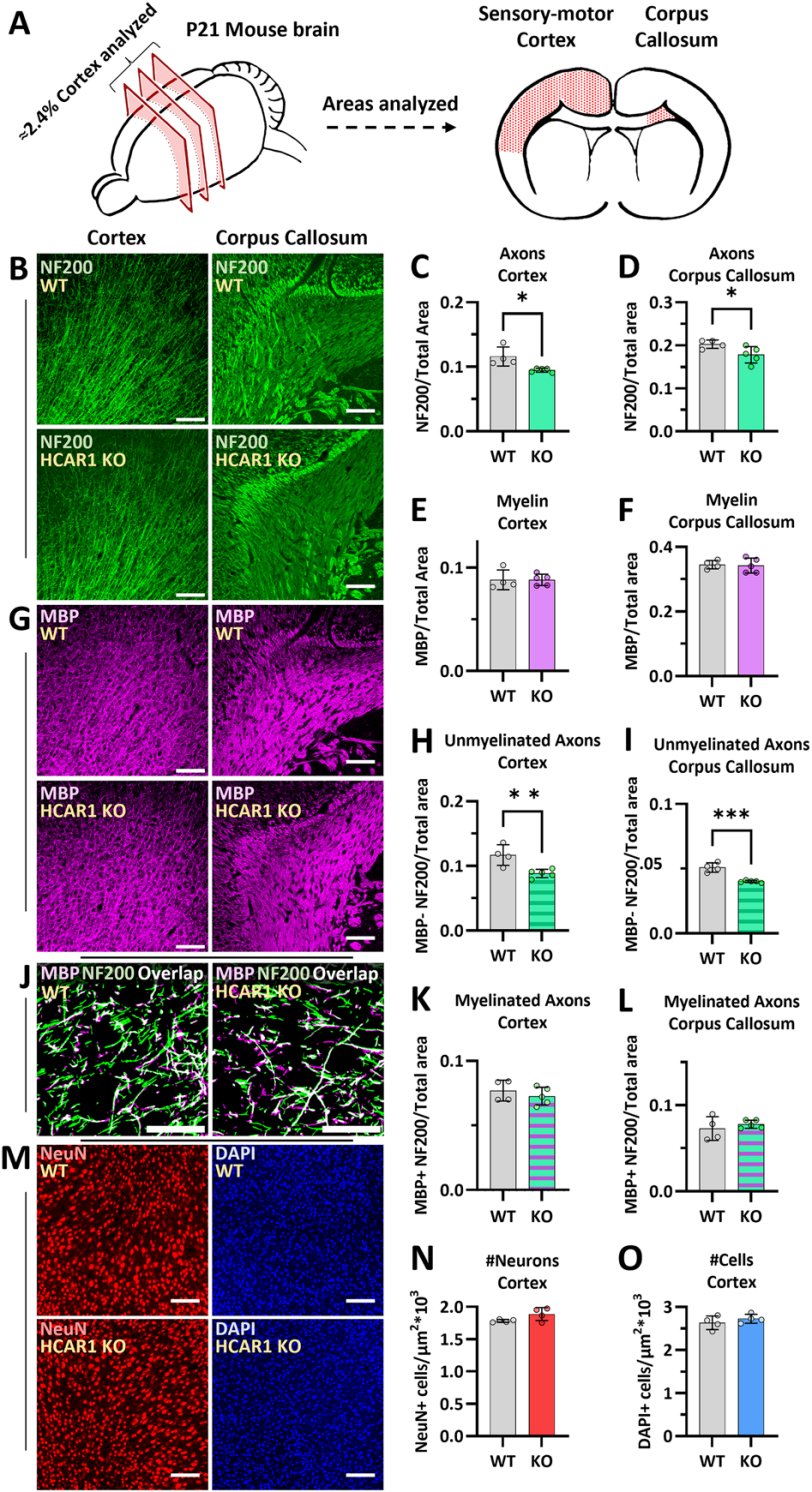
Young HCAR1 KO mice have reduced axonal area in the cortex and corpus callosum. ***A***, Illustration of the coronal sections and areas analyzed (in red). ***B***, ***G***, Example images of cortex and corpus callosum immunostained with the axonal marker NF200 and the myelin marker MBP from WT and HCAR1 KO mice. The top of the cortex images is toward the cortical surface and the bottom of the images is toward the corpus callosum (hence higher density of axons and myelin in the bottom of the image). ***C***, ***D***, Average axonal area in cortex (*p* = 0.015, *n* = 4–5) and corpus callosum (*p* = 0.027, *n* = 4–5) of HCAR1 KO and WT mice. ***E***, ***F***, Average myelin area (cortex *p* = 0.83, corpus callosum *p* = 0.83). ***H***, ***I***, Average area of unmyelinated axons in cortex (*p* = 0.008) and corpus callosum (*p* < 0.001). ***J***, Segmented images of cortical NF200 and MBP showing overlapping structures in white from the cortex. ***K***, ***L***, Average area of myelinated axons in cortex (*p* = 0.42) and corpus callosum (*p* = 0.46). ***M***, Images from the cortex in sections stained with the neuronal marker NeuN and the nuclear marker DAPI. ***N***, ***O***, Average number of cortical neurons and nuclei per area (NeuN *p* = 0.09, DAPI *p* = 0.35, *n* = 4–5). Scale bars: 100 μm, except in ***J*** where scale bar is 50 μm. *p* values from unpaired *t* test. Error bars = SD. Extended Data Figure 1-1 supports this figure.

To test whether HCAR1 influences axonal development and myelination in the brain, we performed immunostaining of coronal sections from the cortex and corpus callosum of HCAR1 KO and WT mice (see illustration of analyzed areas in Fig. 1*A*). We chose to investigate P21 mice, an age at the end of an axonal and synaptic growth spurt, where 90–95% of the brains volume has formed, and that corresponds roughly to 2–3 human years (Semple et al., 2013). In the cortex of HCAR1 KO mice, the axonal area was reduced by 18% relative to WT (%NF200/total area: WT 11.6 ± 1.5, KO 9.5 ± 0.3, *p* = 0.015; Fig. 1*B*,*C*). In the corpus callosum (CC), the axonal area was reduced by 12% in the HCAR1 KO (WT 20.3 ± 1.0, KO 17.8 ± 1.9, *p* = 0.027; Fig. 1*B*,*D*). We did not observe a statistically significant difference in myelin area between HCAR1 KO and WT in either the cortex or CC (%MBP/total area, cortex: WT 29.6 ± 2.5, KO 29.3 ± 2.1, *p* = 0.826. CC: WT 34.5 ± 1.3, KO 34.2 ± 2.3, *p* = 0.823; Fig. 1*E–G*), a somewhat surprising finding given the known correlation between axonal size and myelination (Gillespie and Stein, 1983). This led us to hypothesize that HCAR1 knock-out could selectively affect unmyelinated axons. To explore this, we performed a colocalization analysis where we excluded axons overlapping with myelin to measure the area of unmyelinated axonal fibers. Indeed, the area of unmyelinated axons was reduced in the HCAR1 KO mice compared with WT whereas there was no statistically significant difference in myelinated axons (%MBP-neg. NF200/total area, cortex: WT 11.7 ± 1.6, KO 8.9 ± 0.6, *p* = 0.008. CC: WT 5.1 ± 0.4, KO 4.0 ± 0.01, *p* = 0.001. %MBP-pos. NF200/total area, cortex: WT 7.7 ± 0.8, KO 7.3 ± 0.7, *p* = 0.43. CC: WT 7.3 ± 1.4, KO 7.8 ± 0.5, *p* = 0.46; Fig. 1*H–L*). To control for the difference in total axonal area between the genotypes, we also quantified the ratio of unmyelinated fibers to the axonal area, which similarly revealed a reduced proportion of unmyelinated axons in HCAR1 KO mice (Extended Data Fig. 1-1*D,E*).

10.1523/ENEURO.0563-24.2025.f1-1 Figure 1-1 A-B: Density (number per mm^2^) of axons and myelin sheaths in the cortex from p21 WT and HCAR1 KO mice counted by computer-automated counter. **C:** Number of cortical NeuN/DAPI cells in p21 mice. **D-E** Ratio of unmyelinated axons in the cortex of p21 mice (p=0.024) and corpus callosum (p=0.001). **F** Example images of image segmentation (Seg.) of area and counting analyses NF200 and MBP and the colocalization showing overlapping structures in white, scale bar 40 µm. Download Figure 1-1, TIF file.

We then explored whether the reduced axonal area in HCAR1 KO mice was due to effects on the number of cortical neurons but found no difference in cortical neuron density (#NeuN+ cells/μm^2^ *10^3^: WT 1.78 ± 0.02, KO 1.89 ± 0.1, *p* = 0.09; Fig. 1*M*,*N*). There was also no difference in the total cell density (#DAPI cells/μm^2^ *10^3^: WT 2.63 ± 0.16, KO 2.73 ± 0.10, *p* = 0.35; Fig. 1*M*,*O*) or in the ratio of NeuN+/DAPI cells (*p* = 0.57; Extended Data Fig. 1-1*C*). Lastly, we carried out an analysis to see if the reduction in axonal area reflected a reduced number of axons using a computer-automated particle analysis of NF200 positive objects (i.e., axons). The average axonal number per area did not differ between HCAR1 KO and WT (#NF200 + objects/mm^2^: WT 44,944 ± 3,946, HCAR1 KO 44,116 ± 1,756, *p* = 0.68; Extended Data Fig. 1-1*A*) and thus, the particle analysis indicates that the number of axons is unaffected by HCAR1 KO and that the lower axonal area rather reflects a diminution of axonal size. There was no difference in the number of myelin sheets per area in the cortex (Extended Data Fig. 1-1*B*).

In sum, these data propose that HCAR1 increases axonal area of unmyelinated axons in developing mice without affecting neuronal density or axonal number.

### G-ratio in the optic nerve from young mice is unaltered by HCAR1 KO

**Figure 2.**
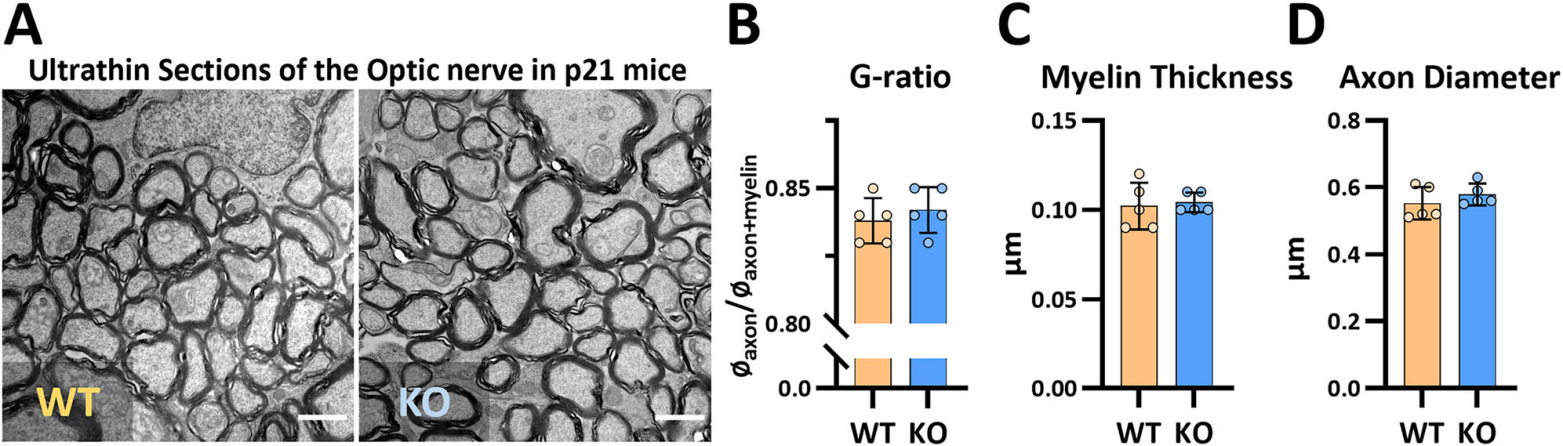
G-ratio and myelin thickness is unaltered in the optic nerve of young HCAR1 KO mice. ***A***, Electron micrographs from coronal ultrathin sections of the optic nerve from P21 WT and HCAR1 KO mice; scale bar, 1 μm. ***B***, G-ratio (relation between myelin and axonal thickness), *p* = 0.47. ***C***, Average myelin thickness, *p* = 0.76. ***D***, Average diameter of myelinated axons, *p* = 0.35. *n* = 5 mice per group.

The immunofluorescence microscopy data suggest that the young HCAR1 KO mice display similar number and size of myelinated axons as the WT. However, due to the limited resolution of light microscopy, we continued with high-resolution transmission electron microscopy to analyze the g-ratio. Here we investigated the optic nerve, a part of the central nervous system that contains solely myelinated axons. The g-ratio describes the relationship between axon size and myelin thickness, and deviations in the g-ratio are thought to play a role in abnormal development and disease (West et al., 2016). There were no differences in g-ratio between the genotypes (WT 0.838 ± 0.008, KO 0.842 ± 0.008, *p* = 0.47; Fig. 2*A*,*B*). Furthermore, there was no difference in the average myelin thickness (WT 102 ± 13 nm, KO 104 ± 6 nm, *p* = 0.76; Fig. 2*A*,*C*) or axonal diameter (WT 552 ± 49 nm, KO 578 ± 33 nm, *p* = 0.35; Fig. 2*A*,*D*). These findings support the immunofluorescence data from the cortex and suggest that under normal conditions, HCAR1 KO does not impact the size of myelinated axons.

### Lactate protects axonal development partly through HCAR1 in prolonged hypoglycemia

**Figure 3.**
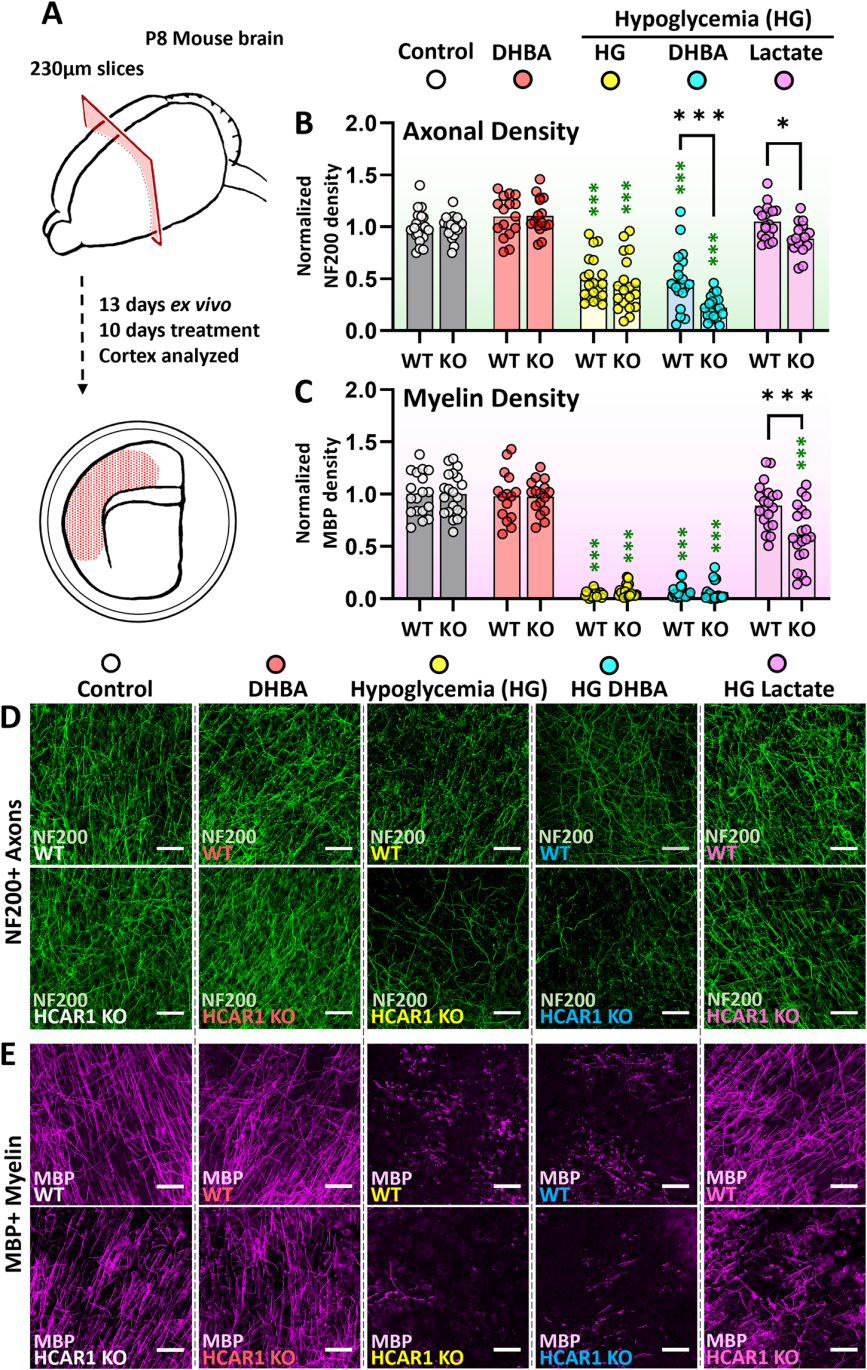
Cortical axonal and myelin area after 10 d HCAR1 stimulation in optimal and hypoglycemic conditions in organotypic brain slice cultures. ***A***, Illustration of the setup for organotypic brain slice culture experiments. The slices were treated with 2 mM of the HCAR1 agonist 3,5-dihydroxy benzoic acid (DHBA) in optimal glucose (41 mM) and hypoglycemic (2,4 mM, HG) and with 20 mM Lactate in HG. ***B***, ***C***, Normalized average axonal and myelin area with different treatment groups. Since WT and KO cultures were made separately, data points were normalized to the average of each genotype's control group. Black *p* value significance * is from Holm–Šidák multiple-comparison test between WT and HCAR1 KO, and the green * from each treatment against their high glucose control (e.g., WT_DHBA vs WT_Control). ***D***, ***E***, Example images of cortex in slices immunostained with the axonal marker NF200 and the myelin marker MBP from WT and HCAR1 KO mice. Scale bar, 50 μm. *n* = 16–20 per group. Extended Data Figure 3-1 supports this figure.

We then sought to investigate whether HCAR1 stimulation could influence axonal development in cortical organotypic brain slice cultures from P8 mice. Brain slices were treated with 2 mM of the HCAR1 agonist 3,5-DHBA (Liu et al., 2012) for 10 consecutive days before fixation on the 13th day ex vivo (corresponding to P21 in vivo). Further, as newborn hypoglycemia is a relatively common perinatal complication thought to have possible long-term consequences on brain maturation (De Angelis et al., 2021) and since lactate is associated with both development (Bauernfeind et al., 2014) and hypoglycemic protection (van Gemert et al., 2022), we wanted to investigate the influence of HCAR1 on axonal and myelin development in a model of perinatal hypoglycemia. For this, we cultured brain slices in low-glucose (2.4 mM) media with or without 20 mM lactate or 2 mM 3,5-DHBA at Days 4–13 in culture. Glucose measurements in the brain slice culture medium showed that glucose concentrations in medium surrounding slices that had been transferred to low glucose was initially higher than 2.4 mM, but the glucose concentration went gradually down after 24 h in low-glucose culture medium (Extended Data Fig. 3-1). The lowest glucose concentration measured was 2.2 mM glucose (72 h after medium change in cultures exposed to DHBA). In high glucose conditions, HCAR1 stimulation did not significantly affect axonal area and there was no difference between the genotypes (%NF200/Control: WT_DHBA_ 109 ± 20, pWT_Control-DHBA_ *p* = 0.291. KO_DHBA_ 111 ± 18, pKO_Control-DHBA_ *p* = 0.209. pDHBA_WT-HCAR1 KO_ *p* = 0.995; Fig. 3*B*,*D*). After hypoglycemia (HG) axonal area was decreased by ∼50% in HCAR1 KO and WT slices (%NF200/Control: WT_HG_ 49 ± 21, KO_HG_ 48 ± 31, pHG_WT-KO_ *p* = 0.803; Fig. 3*B*,*D*). Earlier reports indicate that lactate can maintain axonal integrity during periods of stress, including hypoglycemia (Brown et al., 2004; Rinholm et al., 2011; Fünfschilling et al., 2012), and is found to increase postinjury axonal regeneration of peripheral nerves (Morrison et al., 2015). Indeed, lactate rescued axonal generation in the hypoglycemic slices, however, to a lesser extent in the HCAR1 KO (%NF200/Control: WT_HG_Lactate_ 105 ± 16, KO_HG_Lactate_ 89 ± 15, pHG_Lactate_WT-KO_ *p* = 0.048; Fig. 3*B*,*D*). The HCAR1 agonist 3,5-DHBA failed to save the axonal loss in hypoglycemia, but slices from HCAR1 KO displayed more loss relative to WT (%NF200/Control: WT_HG_DHBA_ 49 ± 28, KO_HG_DHBA_ 22 ± 11, pHG_DHBA_WT-KO_ *p* < 0.001; Fig. 3*B*,*D*). Together these data indicate that the protective effect of lactate on axonal integrity can be enhanced by HCAR1 activity.

10.1523/ENEURO.0563-24.2025.f3-1 Figure 3-1 A: Timeline for slice culture experiments showing start of low glucose treatment, frequency of culture medium changes (pink circles) and timing of glucose measurements at 24, 48 and 72 hours after medium change (blue, yellow and green triangles, respectively). **B-C:** Glucose concentrations in normal (high glucose) culture medium (B) and low glucose medium (C). Glucose was measured in the medium before it was added to organotypic slices (0h; low green), and then 24 (blue), 48 (yellow) and 72 hours (dark green) after medium change. Data from 24, 48 and 72 hours are shown as average (±SD) from two wells. The data are from measurements with Glucose-Glo assay (Promega). Similar results were obtained with an i-STAT1 analyzer (Abbott). Download Figure 3-1, TIF file.

### Lactate-mediated rescue of myelination in hypoglycemia is partly HCAR1 dependent

The myelin-producing oligodendrocytes are even more sensitive to glucose deprivation than neurons. However, lactate can rescue myelination in hypoglycemia (Rinholm et al., 2011). To explore the possible effects of HCAR1 in myelination during hypoglycemia, the organotypic brain slices were labeled with MBP. As with axons, HCAR1 stimulation with 3,5-DHBA did not significantly alter myelination in high glucose conditions in either genotype (%MBP/Control: WT_DHBA_ 98 ± 24, pWT_Control-DHBA_ *p* = 0.431. KO_DHBA_ 98 ± 16, pKO_Control-DHBA_ *p* = 0.734. pDHBA_WT-HCAR1 KO_ *p* > 0.99; Fig. 3*C*,*E*). During prolonged hypoglycemia (HG), myelin area plummeted by >90% in both the HCAR1 KO and WT slices (%MBP/Control: WT_HG_ 4.3 ± 2.8, pWT_Control-HG_ *p* < 0.001. KO_HG_ 7.1 ± 6.0, pKO_Control-HG_ *p* < 0.001. pHG_WT-HCAR1 KO_ *p* = 0.977; Fig. 3*C*,*E*). While lactate could completely rescue myelination in hypoglycemia in slices from WT mice, the HCAR1 KO slices failed to fully restore myelination (%MBP/Control: WT_HG_Lactate_ 89 ± 22, KO_HG_Lactate_ 61 ± 28, pHG_Lactate_WT-KO_ *p* < 0.001; Fig. 3*C*,*E*). However, the HCAR1 agonist 3,5-DHBA did not alter myelination in hypoglycemic slices of either genotype (%MBP/Control: WT_HG_DHBA_ 6.4 ± 6.3. KO_HG_DHBA_ 6.3 ± 8.8, pHG_DHBA_WT-KO_ *p* > 0.99; Fig. 3*C*,*E*). This could possibly be explained by the near total loss of myelin in hypoglycemia (i.e., there was nothing left to rescue). As with the axonal data, these results indicate that HCAR1 can help rescue myelination during hypoglycemia.

### HCAR1 KO appears to increase lactate transport and alter pH buffering in acute cortical brain slices

**Figure 4.**
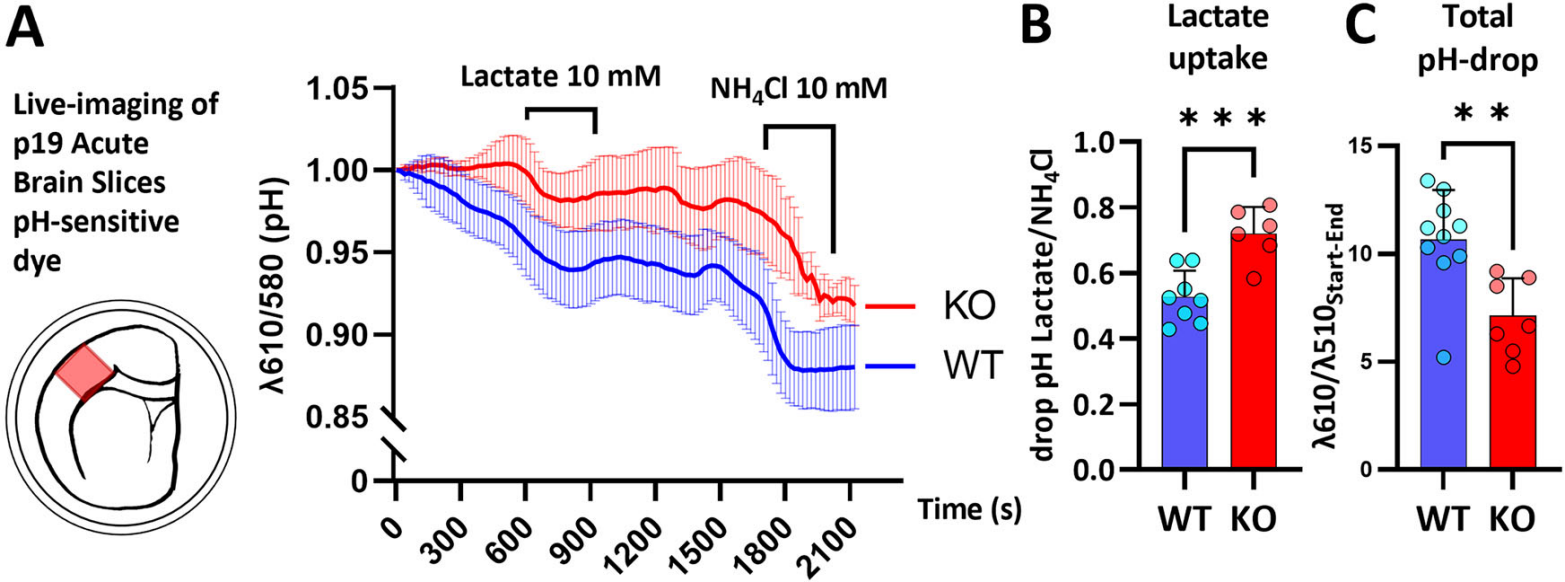
pH imaging in acute cortical brain slices indicates altered lactate uptake in HCAR1 KO mice. ***A***, Illustration and averaged trace (± SD) from acute cortical brain slices loaded with intracellular pH-sensitive dye (SNARF-1 AM), values represent the ratio of emitted wavelengths λ610 and λ580 where decreasing values represent a drop in intracellular pH. Slices were treated with 10 mM lactate for 5 min and lactate uptake was defined as the drop in λ610/λ580 from the expected trajectory during lactate treatment over the drop in control treatment with NH_4_Cl (***B***), *p* < 0.001, *n* = 6–8. ***C***, Graph showing the overall drop in pH from start to end of experiment, *p* = 0.02.

Lactate transport across the cell membrane is crucial for proper metabolic function. As we found that hypoglycemic WT brain slices were rescued by lactate to a larger degree than HCAR1 KO slices, we wanted to test whether HCAR1 can influence the lactate uptake into cells. Lactate flux across cell membranes is facilitated by monocarboxylate transporters, which cotransport lactate and H^+^ and can therefore be monitored by intracellular pH change (Meredith et al., 2002; Rinholm et al., 2011). We performed timelapse confocal imaging with the pH-sensitive ratiometric dye SNARF-1 AM on acute cortical brain slices from P19 HCAR1 KO and WT mice. Application of 10 mM lactate induced a fall in pH in both genotypes, indicating cellular lactate uptake. However, in HCAR1 KO slices the pH drop was larger than in WT (pH drop Lactate/NH_4_Cl: WT 0.53 ± 0.08, KO 0.72 ± 0.08, *p* < 0.001; Fig. 4*A*,*B*), indicating that cells lacking HCAR1 have a higher lactate uptake. Another observation was an apparent difference in pH buffering between HCAR1 KO and WT slices. When imaging acute brain slices over several tens of minutes, a common phenomenon is the gradual acidification of the tissue (Rinholm et al., 2011). In our experiments, the pH tended to fall less over time in KO slices compared with WT, leading to a higher pH at the end of the experiment (% Total pH drop: WT 10.7 ± 2.3, KO 7.1 ± 1.8, *p* = 0.02; Fig. 4*C*).

## Discussion

Here we present evidence of HCAR1 involvement in axonal development within the rodent cortex and corpus callosum. We report that P21 HCAR1 KO mice exhibit reduced size of unmyelinated axons in the cortex and corpus callosum. Additionally, our ex vivo model of neonatal hypoglycemia shows that lactate protects axonal and myelin development and that HCAR1 plays a role in this protective effect. Furthermore, live pH imaging indicates that intracellular lactate uptake is altered in brain slices from HCAR1 KO mice. Our findings reveal new roles of the lactate receptor HCAR1 in brain development and as a therapeutic target for neonatal hypoglycemia.

In the P21 HCAR1 KO mice, axonal area was reduced in the cortex and the corpus callosum (Fig. 1). A recent study found that HCAR1 increases both axonal size and number in the retinothalamic CNS (Laroche et al., 2021). Our analysis of the cortex, however, showed no reduction in the average number of axons in HCAR1 KO (Extended Data Fig. 1-1) suggesting that the observed reduction in area is due to effects on axonal size alone. It should be noted that the particle analysis used has a rather low spatial resolution and counts overlapping axons as one. However, if the difference between the genotypes was due to overlapping axons, this would cause the objects analyzed to have an increase in the parameter circularity, but this was not the case in our dataset (data not shown). Additionally, the analysis is relatively high throughput, with 126,450 to 146,400 objects, i.e., axons, analyzed per mouse.

Myelination of CNS axons is dependent on the neuronal subtype and the axonal diameter, and the thickness of myelin sheaths increase with axonal diameter (Gillespie and Stein, 1983; Call and Bergles, 2021). Thus, we anticipated that the reduced axonal area would correspond with fewer or smaller myelin sheaths. Surprisingly, we observed no difference in the myelin area between the HCAR1 KO and WT (Fig. 1). Furthermore, there was no difference in g-ratio or average myelin thickness in optic nerve axons (Fig. 2). However, the electron microscope analysis of g-ratio was only performed in the optic nerve, and not in the cortex where the immunofluorescence data was obtained. So, it is possible that a change in g-ratio does exist in the cortex. Nevertheless, our data suggest that unmyelinated axons are preferentially affected by the HCAR1 KO and indeed, using object-based colocalization analysis, we could determine that both the proportion of unmyelinated axons and the total area of unmyelinated axons where lower in the HCAR1 KO, while there was no difference in the area of myelinated axons (Extended Data Fig. 1-1). Unmyelinated axons have higher energetic costs of maintaining action potentials than myelinated axons (Hartline and Colman, 2007) and are thus more vulnerable to changes in energy supply (Nave et al., 2023). In vitro studies suggest that HCAR1 is involved in the regulation of energy metabolism (Jin et al., 2022). Thus, it is possible that a dysregulation of energy metabolism in HCAR1 KO causes the specific loss of unmyelinated axons, but more studies are needed to test this. It is reported that, dependent on neuronal type, ∼30–90% of cortical axons (Call and Bergles, 2021) and ∼80% in the axons in the corpus callosum (Reeves et al., 2012) are unmyelinated. Unmyelinated axons propagate signals at a much lower pace compared with myelinated axons and are involved in neuromodulation and slower processes like chronic pain and autonomic nervous system functions where rapid responses are less critical (Kandel et al., 2021). Thus, the fact that we only saw a difference in unmyelinated axons could help explain why there are no obvious phenotypic characteristics in motor function, daily activities, or physical properties of the HCAR1 KO mouse (own unpublished observations, de Castro Abrantes et al., 2019). Interestingly, a recent study reported that HCAR1 KO mice exhibit differences in certain social, anxiety, and, repetitive behaviors in an autism-like manner (Mohammad Nezhady et al., 2023). Thus, for the first time documenting possible behavioral implications of HCAR1 KO that can be linked to its reported effects on neuronal firing (Bozzo et al., 2013; de Castro Abrantes et al., 2019; Briquet et al., 2022) and, as we show here, axonal development. Worth mentioning in the context of our axonal findings is that our analyses do not include all axons; The marker we used, NF200, is also known as heavy chain neurofilament and, hence, is not expressed in every axon. Furthermore, NF200 is expressed later in development compared with the other neurofilaments, light and medium chain (Julien et al., 1986). In conclusion, our data suggest that HCAR1 affects axonal development, painting a picture of HCAR1 modulating neuronal activity not only through alterations in neurotransmission, but also through structural impacts on neuronal axons.

Previous to our report, one study has shown that HCAR1 promotes axonogenesis in the retinal CNS in vivo and in vitro (Laroche et al., 2021). Contrary to their findings in retinal explants, stimulation with the HCAR1 agonist 3,5-DHBA at high glucose concentrations did not affect axonal growth in our cortical organotypic brain slice cultures (Fig. 3). This discrepancy suggests regional differences, but there are also significant distinctions in the experimental setups which may affect the results. In the present study, we looked at axonal growth after prolonged HCAR1 activation in an ex vivo model at postnatal period P12–21, whereas Laroche et al., 2021 used explants from fetal E15 mice that were treated one or 15 h before analysis. Furthermore, it is conceivable that HCAR1 gets sufficient stimulation from endogenously produced lactate by glial cells (Pellerin and Magistretti, 1994; Fünfschilling et al., 2012; Lee et al., 2012), thereby making stimulation with 3,5-DHBA superfluous. Unfortunately, WT and HCAR1 KO organotypic cultures were produced at different time points and the data were normalized to the experiment control due to significant variation between experiments. Hence, untreated controls could not be directly compared between WT and KO.

Here we used cortical organotypic brain slice cultures in a medium containing 2.4 mM glucose as a model of neonatal hypoglycemia. However, the glucose measurements showed that the actual glucose concentration was higher at the beginning of the low-glucose treatment. This could be due to leftover high glucose medium in the brain slice and insert membrane holding the slice. It is also possible that glucose taken up into the cells during exposure to high glucose was released when the slices were placed in low-glucose medium, since glucose transporters facilitate diffusion of glucose, with the concentration gradient, across the membrane. The brain slices were harvested at p8, an age approximately corresponding to the early period of the human infant term (Hagberg et al., 2002), stabilized for 4 d before 10 d of hypoglycemia. Physiological plasma glucose concentrations are within the range of 4–5.5 mM but extracellular brain glucose concentrations are significantly lower and reported between 0.7 and 2.5 mM (Handbook of Neurochemistry and Molecular Neurobiology: Sensory Neurochemistry | SpringerLink, 2007). Although culture media is designed to mimic the extracellular milieu, the glucose concentrations used to achieve optimal organotypic slice cultures are highly hyperglycemic (Stoppini et al., 1991; De Simoni and MY Yu, 2006; Humpel, 2015), with our slice medium containing as much as 41 mM glucose (since, in addition to the added glucose, MEM, EBSS, and horse serum also contain glucose). It is common to use hyperglycemic medium in all cell culture systems but the thickness of organotypic slices means that nutrients need to traverse a longer distance to reach the cells at the core of the slice in comparison with dissociated or single cell layer culture systems. Consequently, the extracellular concentration of glucose is somewhat lower in the middle of the slice than at the surface (Tekkök et al., 2002). Looking at the severe effect on myelination and axonal generation of culturing in low-glucose medium (Fig. 3), we believe this model is most translatable to severe prolonged neonatal hypoglycemia. Lactate has proven protective in adult diabetic hypoglycemia (Maran et al., 1994; Veneman et al., 1994; King et al., 1997; King et al., 1998; Maran et al., 2000; De Feyter et al., 2013; Wiegers et al., 2019); however, studies from neonatal hypoglycemia are limited. Lactate is well recognized as an energy substrate for neurons (Schurr, 2006; Fünfschilling et al., 2012) and can metabolically support neurons and oligodendrocytes in neonatal hypoglycemia in vitro (Rinholm et al., 2011; Harris et al., 2015), an attribute clearly supported by our results (Fig. 3). Hence, a large part of the lactate-restoring effect of axons and myelin is metabolic and not dependent on HCAR1. Furthermore, our data propose that HCAR1 plays a role in lactate protection in hypoglycemia as lactate treated HCAR1 KO slices had poorer axonal and myelin development relative to WT. The axonal area in 3,5-DHBA-treated HCAR1 KO slices was less than in the WT; however, there was seemingly no difference between the untreated hypoglycemic and 3,5-DHBA-treated WT slices. It is unclear why application of 3,5-DHBA would reduce the axon area in KO slices, and unspecific drug effects cannot be ruled out.

It should be noted that the components of the culture medium could have undesired effects on neuronal signaling and axonal growth. For instance, penicillin can work as a GABA(A) receptor antagonist (Rossokhin et al., 2014), and nystatin can influence axonal outgrowth (Roselló-Busquets et al., 2020). However, the concentrations of penicillin and nystatin used in our cultures were ∼1,000 times lower than the concentrations with demonstrated effects on cells. While such effects cannot be completely ruled, they should be similar across our experiments as equal concentrations were used throughout.

Lactate's function as an energy substrate and signaling molecule makes the knowledge of lactate transport across cell membranes crucial for a deeper understanding of its function. Here we present data suggesting that lactate uptake in cortical brain cells is altered by HCAR1. Our findings indicate that intracellular lactate uptake is increased in HCAR1 KO cells (Fig. 4), which is contrary to reports in cancer biology, where monocarboxylate transporters (MCTs) and lactate uptake is found downregulated in HCAR1 silenced cells (Roland et al., 2014; Tafur et al., 2019; Longhitano et al., 2022). MCTs shift lactate and H^+^ across membranes passively according to the concentration gradient. Therefore, we see three major ways in which HCAR1 KO could influence MCT mediated lactate transport: (1) regulation of MCT expression on the cell membrane, (2) regulation of intracellular lactate metabolism, and/or (3) alteration of the concentration gradient of H^+^ across cell membranes, e.g., by altering systems like Na^+^/H^+^ and HCO_3_^−^/Cl^−^ pumps or intracellular biochemical reactions (Ruffin et al., 2014). Although we have not found previous reports on brain HCAR1 and lactate uptake, one study of retinal glial cell cultures found that HCAR1 activation increased lactate and glucose consumption thus reducing lactate efflux (Vohra et al., 2019). Complicating the translatability of this finding, the same group could not replicate this finding in a multicellular system of retinal explants (Vohra et al., 2022). Further, a study in cancer cells found that HCAR1 increased glycolysis and reduced oxidative phosphorylation, whereas HCAR1 KO led to lowered glycolysis and higher oxidative phosphorylation (Jin et al., 2022). This could fit with our data showing higher lactate uptake in HCAR1 KO slices, which, according to this regime, would have less endogenous lactate production and more lactate consumption through oxidative phosphorylation. We can also not rule out the possibility of HCAR1 regulating the expression of lactate transporters. This should be tested in future studies. Interestingly, another means of lactate transport has been reported in astrocytes through an ion channel permeable to lactate (Sotelo-Hitschfeld et al., 2015). They found that upon membrane depolarization or increased extracellular K^+^, lactate is released through this ion channel and that the lactate efflux is sustained by a positive feedback loop that can uphold flow even against the concentration gradient. Furthermore, depolarization or K^+^ could increase glucose metabolism in astrocytes surrounded by active neurons (Ruminot et al., 2019). HCAR1 activation is found to inhibit spontaneous neuronal firing (Bozzo et al., 2013; Briquet et al., 2022). One could hypothesize that HCAR1 activation leads to reduced extracellular K^+^ near astrocytes and that this will cause reduced extracellular glucose- or glycogen-derived lactate from astrocytes available for neuronal uptake. This hypothesis could fit our data as lactate would cause an increased production of glycogen-derived lactate which would exit astrocytes and enter other brain cells through MCTs in HCAR1 KO slices due to the expected higher spontaneous neuronal activity upon lactate exposure.

Lastly, we saw a tendency of HCAR1 KO slices to better withstand the gradual acidification that occurs in acute brain slices (Fig. 4). This could be due to better buffer capacity of HCAR1 KO cells or by reduced lactate consumption that in turn leads to efflux of lactate with H+. Both these scenarios would be a source of misinterpretation of HCAR1 KO cells to have an increased capacity for lactate uptake as concentrations of intracellular lactate and/or H+ would be different at the time of lactate treatment.

In conclusion, our findings strongly indicate HCAR1 involvement in axonal development and suggest that lactate, acting partly through HCAR1, exerts protective effects in experimental neonatal hypoglycemia. This study is largely descriptive and further investigations into the functional mechanisms of these findings is warranted to advance understanding in this area.

## Synthesis

Reviewing Editor: Deanna Smith, University of South Carolina Decisions are customarily a result of the Reviewing Editor and the peer reviewers coming together and discussing their recommendations until a consensus is reached. When revisions are invited, a fact-based synthesis statement explaining their decision and outlining what is needed to prepare a revision will be listed below. The following reviewer(s) agreed to reveal their identity: NONE.

While the reviewers found several strengths in the work some issues need to be addressed before the study is ready for publication.

1) The images of KO cortex in figures 1B and 1G show an area with modest axonal and myelin loss adjacent to an area with more widespread loss. Either a more representative image should be shown or the authors should explain the more regional axonal and myelin changes.

2) The finding that HCAR1 KO mice have fewer nonmyelinated axons is presented in the abstract, and is therefore a major finding of the study, yet the data supporting this claim is presented only in the supplement. No raw data is presented supporting this summary data. Summary figure 1 should be included in body of the paper with raw images supporting the summary data.

3) Images should be provided showing what NF200 structures were considered by Image J analysis to be axons.

4) Insufficient information is provided about experimental design. Stereotaxic coordinates of ROI should be provided. Dilutions of the antibodies should be provided. Examples of colocalization analysis should be shown to further explain how myelinated axons were excluded to measure the area of unmyelinated axonal fibers.

5) Figure 2 performs G-ratio measurements in optic nerve rather than corpus callosum. The provided rationale for this study is weak. Many studies have examined G-ratio in corpus callosum. Ultrastructural studies in corpus callosum should be done for they can be directly compared to the immunohistology study in figure 1 as well as directly assess the issue of axonal number vs area.

6) The author should discuss the potential confounds that slices are cultured in penicillin which is a GABA-A antagonist as well as a antimicrobial. The authors should also discuss the potential confound that Nystatin can alter axonal outgrowth (Roselló-Busquets C, et al.. Nystatin Regulates Axonal Extension and Regeneration by Modifying the Levels of Nitric Oxide. Front Mol Neurosci. 2020 Apr 3;13:56).

7) The authors should discuss why the majority of lactate restoration of myelin is not HCAR dependent (Figure 3)?

8) Information presented in the discussion is better included in the introduction. "it is recommended that plasma glucose levels in the newborn infant stay above 3.3 mM with a somewhat wider tolerance during the first 48 hours after birth where many infants experience physiological transient hypoglycemia (Thornton et al., 2015). Here we used cortical organotypic brain slice cultures maintained in a medium containing 2.4 mM glucose as a model of neonatal hypoglycemia."

9) A large number of axons were counted and studied, and EM was performed to confirm the G-ratio, however, the descriptive study does not explain why the axonal size is lower for this proportion of neurons. Can the authors speculate more on why the normal number of neurons are present in a myelinated form, and why only a subset are smaller and unmyelinated?. Also, were these smaller diameter neurons in a certain portion of that brain region? Could these neurons differ in their expression of other transporters for lactate?

10) The 36 mM glucose used for high glucose/normoglycemia is very high. If medium is replaced every 2-3 days the authors should validate the glucose levels on day 2 or 3. Medium can be sampled and read on a hand held glucometer.

## Author Response

Dear Editor, Here we submit a revised version of our manuscript (eN-NWR-0563-24) entitled "Lactate receptor HCAR1 affects axonal development and contributes to lactate's protection of axons and myelin in experimental neonatal hypoglycemia".

We have amended the manuscript to answer the requests made by the reviewers. Below, I have included a detailed response to the reviewers' comments. The conclusions of the manuscript remain the same after the new amendments.

We hope that our manuscript can now be accepted for publication in eNeuro.

On behalf of the authors, Johanne Egge Rinholm Response to reviewers comments. Please note that changes are highlighted in yellow in the manuscript.

1) The images of KO cortex in figures 1B and 1G show an area with modest axonal and myelin loss adjacent to an area with more widespread loss. Either a more representative image should be shown or the authors should explain the more regional axonal and myelin changes.

We acknowledge the concern regarding apparent regional differences in axonal and myelin density in Figures 1B and 1G. The contrast in previous images may have unintentionally exaggerated these differences. In the revised manuscript, we have replaced the images with more representative sections and adjusted the contrast to more accurately depict anatomical variation, specifically, the normal lower density of myelinated axons in superficial cortical layers.

2) The finding that HCAR1 KO mice have fewer nonmyelinated axons is presented in the abstract, and is therefore a major finding of the study, yet the data supporting this claim is presented only in the supplement. No raw data is presented supporting this summary data. Summary figure 1 should be included in body of the paper with raw images supporting the summary data.

We agree. As requested, we have now included representable segmented images that were used for quantification of myelinated and unmyelinated axons (Fig. 1J). The corresponding bar plots showing the quantified unmyelinated and myelinated axons have been moved from supplementary to Fig 1 (Fig. 1H, I, K, L).

3) Images should be provided showing what NF200 structures were considered by Image J analysis to be axons.

To show this, we have now included raw images of NF200 labeled axons and MBP labeled myelin next to the segmented images showing the structures identified by Image J as axons and myelin (Extended data, Figure 1-1 F).

4) Insufficient information is provided about experimental design. Stereotaxic coordinates of ROI should be provided. Dilutions of the antibodies should be provided. Examples of colocalization analysis should be shown to further explain how myelinated axons were excluded to measure the area of unmyelinated axonal fibers.

We have added the missing experimental parameters in Methods. Stereotactic coordinates have been provided under "Perfusion fixation and cryosectioning" and "Organotypic brain slice cultures". Antibody dilutions are given under "Immunohistochemistry". Examples of colocalization analysis has been added to Figure 1 and Extended data Figure 1-1 as explained in points 2 and 3 above.

5) Figure 2 performs G-ratio measurements in optic nerve rather than corpus callosum. The provided rationale for this study is weak. Many studies have examined G-ratio in corpus callosum. Ultrastructural studies in corpus callosum should be done for they can be directly compared to the immunohistology study in figure 1 as well as directly assess the issue of axonal number vs area.

Although we agree that corpus callosum analysis would have provided a more direct comparison, we did not have access to appropriately processed tissue. We therefore used the optic nerve, which enables robust cross-sectional g-ratio analysis. The limitation of this substitution is now explicitly addressed in the revised discussion.

6) The author should discuss the potential confounds that slices are cultured in penicillin which is a GABA-A antagonist as well as a antimicrobial. The authors should also discuss the potential confound that Nystatin can alter axonal outgrowth (Roselló-Busquets C, et al.. Nystatin Regulates Axonal Extension and Regeneration by Modifying the Levels of Nitric Oxide. Front Mol Neurosci. 2020 Apr 3;13:56).

The concentrations of penicillin and nystatin used in our experiments are about 1000 times lower than those used to give the effects mentioned above. But since we can still not rule out the possibility of such effects occurring, we have included this caveat in the discussion.

7) The authors should discuss why the majority of lactate restoration of myelin is not HCAR dependent (Figure 3)? We believe this is due to the metabolic effects of lactate. We have not put more emphasis on this point in the discussion.

8) Information presented in the discussion is better included in the introduction. "it is recommended that plasma glucose levels in the newborn infant stay above 3.3 mM with a somewhat wider tolerance during the first 48 hours after birth where many infants experience physiological transient hypoglycemia (Thornton et al., 2015). Here we used cortical organotypic brain slice cultures maintained in a medium containing 2.4 mM glucose as a model of neonatal hypoglycemia." We agree that neonatal hypoglycemia should be presented in the introduction. We have now included a paragraph about this in the introduction, and we have moved the part mentioned by the reviewer, up to this paragraph.

9) A large number of axons were counted and studied, and EM was performed to confirm the G-ratio, however, the descriptive study does not explain why the axonal size is lower for this proportion of neurons. Can the authors speculate more on why the normal number of neurons are present in a myelinated form, and why only a subset are smaller and unmyelinated?. Also, were these smaller diameter neurons in a certain portion of that brain region? Could these neurons differ in their expression of other transporters for lactate? The smaller diameter neurons were not localized to a specific portion of the observed brain regions. Unmyelinated axons have higher energy expenses of firing action potentials than myelinated axons. This makes them more vulnerable to fluctuations in energy accessibility. In vitro studies have suggested a role for HCAR1 in regulating energy metabolism. Thus, a dysregulation of energetic pathways in HCAR1 KO could potentially harm unmyelinated axons more than myelinated ones. A difference in the expression of MCTs or other lactate transporters cannot be ruled out. We have now included these points in the discussion.

10) The 36 mM glucose used for high glucose/normoglycemia is very high. If medium is replaced every 2-3 days the authors should validate the glucose levels on day 2 or 3. Medium can be sampled and read on a hand held glucometer.

Glucose levels were measured at 0h, 24h, 48h, and 72h post-medium replacement using both a Glucose-Glo{trade mark, serif} bioluminescent assay and an iSTAT-1 glucose meter (Extended data, Figure 3-1). These measurements showed an unexpected rise at 24h, likely due to residual high glucose in membranes or culture inserts, or release of glucose from the cells-followed by a decline in the low glucose condition. We acknowledge that 36-41 mM glucose is very high. This is still commonly used in cell culture. This point is mentioned in the discussion.
