# Supplementary figures and images for: Lactate Receptor HCAR1 Affects Axonal Development and Contributes to Lactate’s Protection of Axons and Myelin in Experimental Neonatal Hypoglycemia
Source: eNeuro. 2025 May 30;12(5):ENEURO.0563-24.2025. doi: 10.1523/ENEURO.0563-24.2025 (PMC12124765; doi:10.1523/ENEURO.0563-24.2025)

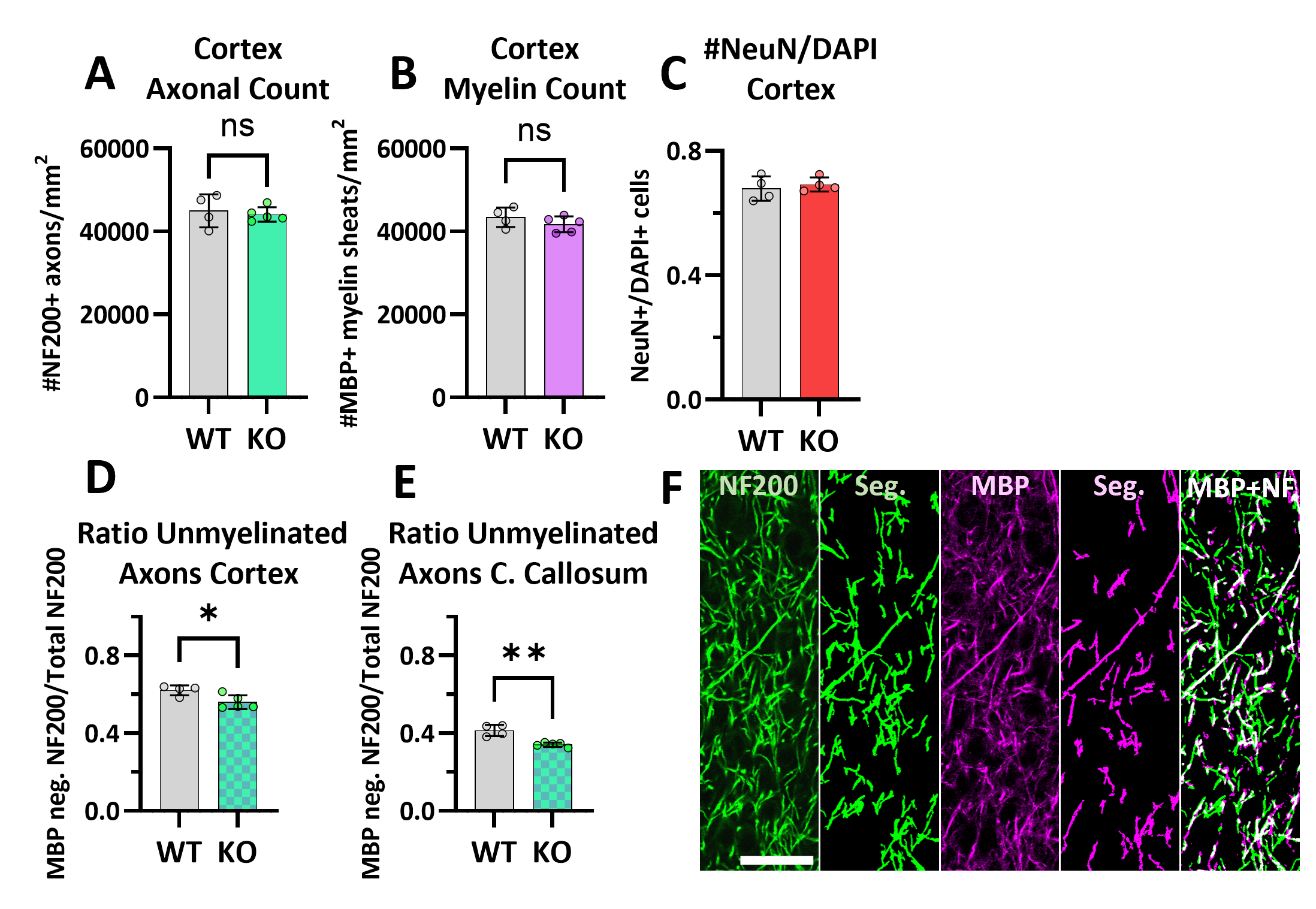

Supplement: Figure 1-1 — A-B: Density (number per mm2) of axons and myelin sheaths in the cortex from p21 WT and HCAR1 KO mice counted by computer-automated counter. C: Number of cortical NeuN/DAPI cells in p21 mice. D-E Ratio of unmyelinated axons in the cortex of p21 mice (p=0.024) and corpus callosum (p=0.001). F Example images of image segmentation (Seg.) of area and counting analyses NF200 and MBP and the colocalization showing overlapping structures in white, scale bar 40 µm. Download Figure 1-1, TIF file. [file eneuro-12-ENEURO.0563-24.2025-s001.tif]

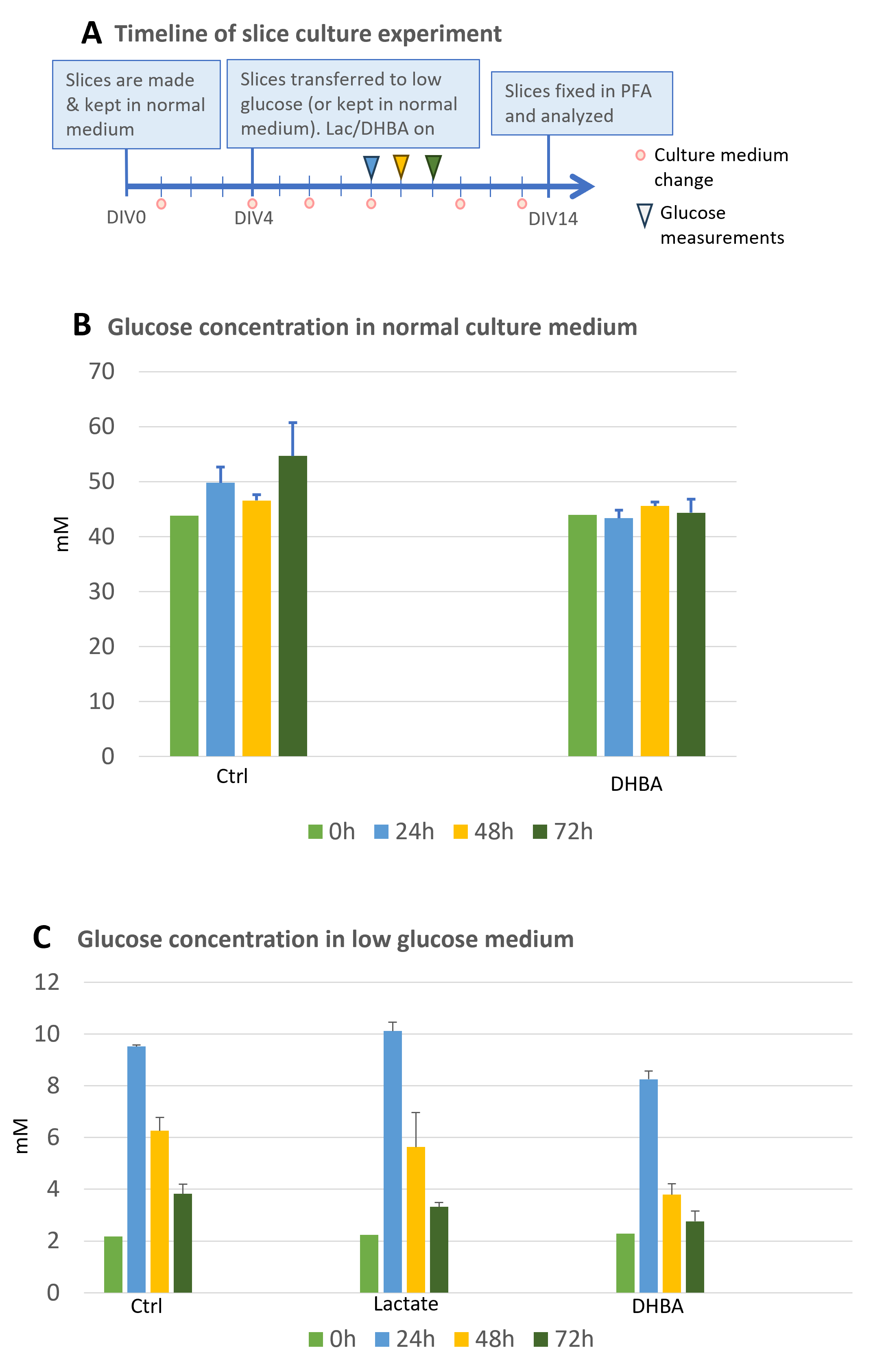

Supplement: Figure 3-1 — A: Timeline for slice culture experiments showing start of low glucose treatment, frequency of culture medium changes (pink circles) and timing of glucose measurements at 24, 48 and 72 hours after medium change (blue, yellow and green triangles, respectively). B-C: Glucose concentrations in normal (high glucose) culture medium (B) and low glucose medium (C). Glucose was measured in the medium before it was added to organotypic slices (0h; low green), and then 24 (blue), 48 (yellow) and 72 hours (dark green) after medium change. Data from 24, 48 and 72 hours are shown as average (±SD) from two wells. The data are from measurements with Glucose-Glo assay (Promega). Similar results were obtained with an i-STAT1 analyzer (Abbott). Download Figure 3-1, TIF file. [file eneuro-12-ENEURO.0563-24.2025-s002.tif]
